# Intravenous-to-oral antibiotic switch therapy: a cross-sectional study in critical care units

**DOI:** 10.1186/s12879-019-4280-0

**Published:** 2019-07-22

**Authors:** Juliano Gasparetto, Felipe Francisco Tuon, Dayana dos Santos Oliveira, Tiago Zequinao, Gabriel Rammert Pipolo, Gabriel Velloso Ribeiro, Paola Delai Benincá, June Alisson Westarb Cruz, Thyago Proenca Moraes

**Affiliations:** 1 School of Medicine, Pontifícia Universidade Católica do Paraná, Curitiba, PR Brazil; 2 Laboratory of Emerging Infectious Diseases, Escola de Medicina, Pontifícia Universidade Católica do Paraná, Rua Imaculada Conceição, 1155, Curitiba, PR 80215-901 Brazil; 3 Business School, Pontifícia Universidade Católica do Paraná, Curitiba, PR Brazil

**Keywords:** Antibiotic, Oral switch, Stewardship, Intensive care unit, Critically ill patients

## Abstract

**Background:**

This study aimed to evaluate the oral switch (OS) stewardship intervention in the intensive care unit (ICU).

**Methods:**

This was a retrospective study with a convenience sample in two Brazilian ICUs from different hospitals in patients with sepsis receiving antibiotic therapy. The stewardship intervention included OS in patients diagnosed with sepsis when clinical stability was achieved. The primary outcome was overall mortality. Other variables evaluated were as follows: cost of antimicrobial treatment, daily costs of intensive care, acute kidney injury, and length of stay.

**Results:**

There was no difference in mortality between the OS and non-OS groups (*p* = 0.06). Length of stay in the ICU (*p* = 0.029) was shorter and acute kidney injury incidence (*p* = 0.032) and costs of antimicrobial therapy (*p* < 0.001) were lower in the OS group.

**Conclusion:**

OS stewardship programs in the ICU may be considered a safe strategy. Switch therapy reduced the cost and shortened the length of stay in ICUs.

**Electronic supplementary material:**

The online version of this article (10.1186/s12879-019-4280-0) contains supplementary material, which is available to authorized users.

## Background

An antimicrobial stewardship program can be defined as the set of actions performed in hospitals for the rational use of antibiotics; reduction of adverse events, dosage errors, and appearance of multiresistant bacteria; and shortening of length of hospital stay. The performance of the multiprofessional team implementing these programs, including clinical pharmacists, physicians and nurses, is associated with a reduction in in-hospital mortality rate [1]. The vast majority of patients receive intravenous (IV) antibiotics during hospitalization due to several factors, including infection severity and low bioavailability of some antibiotics. In hospitalized patients, after a period of 72 h of initial stabilization, 83% of patients would be unnecessarily receiving IV antibiotics, increasing the treatment costs by 200% in the case of some classes of antibiotics and prolonging hospital stay [2].

Managed antimicrobial administration programs shorten the length of hospital stay and reduce costs associated with the use of these medications. Although the literature is consistent with the transition from IV to oral antibiotics in hospitalized patients, data on a similar approach in critically ill patients are inadequate [3]. Some concerns on changing the route of administration are described: poor intestinal absorption, different serum levels, and patients in intensive care units (ICUs) [4]. Although there are some concerns, some classes of antimicrobials have oral bioavailability similar to that in IV administration, such as quinolones, even when administered in critically ill patients [5].

The switch from IV to oral route may have some benefits, such as early discharge, decreased risk of bacteremia, reduced use of venous access and incidence of thrombophlebitis, and reduced cost of treatment [6]. For example, the unit cost of a ciprofloxacin tablet is $0.053, while that of the IV formulation is $3.64, which may represent a significant difference in low- and medium-income countries, such as Brazil [7]. Considering all these aspects, we hypothesized that IV-to-oral antibiotic switch therapy in critical care units can be a safe and cost-effective approach in selected patients.

This study aimed to evaluate the safety, mortality, and economic outcome of IV-to-oral antibiotic switch therapy in critical care units.

## Methods

### Study design and setting

This was a retrospective study with a convenience sample of critically ill patients with sepsis who received antibiotic therapy in two ICUs of different Brazilian hospitals from January 2016 to March 2018. Patient inclusion was performed at two hospitals in the city of Curitiba: a 207-bed public school hospital (Hospital Universitário Cajuru), whose ICU is a referral center for the care of patients with trauma, and a 210-bed hospital (Hospital Santa Casa de Curitiba), with an ICU specializing in clinical and surgical care. The local ethics committee (Comitê de Ética em Pesquisa da Pontifícia Universidade Católica do Paraná) approved this study (committee’s reference number = 74844017800000020). No administrative permissions were required to access the raw data. The requirement of informed consent was waived by the institutional review board. The STROBE checklist is presented as Additional file 1.

The primary outcome was safety (global mortality) in the IV-to-oral switch (OS) groups compared to the group of patients who did not switch to oral administration. The two groups were formed in accordance with the stewardship intervention decision described below and possibility of OS.

### Inclusion and exclusion criteria

The inclusion criteria were as follows: age > 18 years, admission to the ICU with the clinical diagnosis of sepsis or septic shock (in accordance with Sepsis-3 criteria), regular oral or enteral feeding at a flow rate > 40 mL/h, availability of a suitable oral dosage form for the prescribed medication, absorption and bioequivalence of the oral dosage form comparable with the parenteral pharmaceutical form, consent provided by the attending team, and at least 24 h of observed clinical improvement. Antimicrobial therapy was administered to all patients with a life expectancy of > 24 h and those for whom treatment was not considered futile. The sample size was determined by convenience.

The exclusion criteria were as follows: fasting, diet intolerance or refusal to receive oral medications, refusal of the attending team or ICU, increased gastrointestinal bleeding, and absence of clinical improvement with IV treatment or worsening in the last 24 h prior to the multiprofessional visit.

### Stewardship program

The stewardship program was initiated in both hospitals by the same team with the aim of promoting the rational use of antimicrobials, including dose adjustment, route of administration, and appropriate selection of treatment regimens. Thus, daily multiprofessional visits were conducted, which included evaluation by an infectious disease specialist, nurse, and clinical pharmacist, who reviewed the cases and suggested to the assistant and intensivist the possibility of changing the route of administration from IV to oral, after 24 h of clinical recovery. An application for consultation of institutional protocols and bioequivalence of medications was available. The attending physician and ICU attendant could modify the route of administration in the case of clinical worsening (oral to IV) at any moment by discussion using a mobile phone with the application WhatsApp, 24 h a day, 7 days per week [8].

Formulary restriction was not used in this group of patients because all of them were included in the sepsis protocol of the hospital. In the sepsis protocol of both hospitals, antibiotic administration is promptly authorized without restriction. Briefly, for sepsis secondary to community pyelonephritis, pneumonia, intra-abdominal infection, and meningitis, ceftriaxone was the first option, including metronidazole for intra-abdominal infection. Cefepime was the first option for community infection in patients who recently used antibiotic or those admitted within < 5 days. For ventilator-associated pneumonia and most hospital infections, amikacin with levofloxacin was the most common option for treatment, in accordance with local epidemiology. Vancomycin is added if presence of methicillin-resistant *Staphylococcus aureus* (MRSA) is suspected. The complete protocol of the hospital is detailed on the following website: www.atbhuc.goodbarber.com. The most common options for OS were amoxicillin (capsules or solution for nasogastric feeding), amoxicillin/clavulanate (capsules or solution for nasogastric feeding), ciprofloxacin (whole or crushed tablets), levofloxacin (whole or crushed tablets), doxycycline (capsules), sulfamethoxazole/trimethoprim (tablets or solution for nasogastric feeding), and metronidazole (whole or crushed tablets). The choice of oral therapy was defined during clinical visit according to the culture results, infection site, organ dysfunction, and possible side effects. OS is part of a more inclusive strategy, and the possible impact of other interventions, such as dose adjustment, infusion duration, antimicrobial combination, and drug interaction, on the evaluated switch cannot be separately analyzed.

The decision on treatment duration is based on the clinical response and infection site. Generally, the duration of ventilator-associated pneumonia treatment is 4–6 days, with the exception of *Pseudomonas aeruginosa* (14 days) [9]. Intra-abdominal and urinary tract infections were also treated in accordance with Infectious Disease Society of America guidelines [10, 11].

### Clinical data

The following clinical and laboratory data were assessed: sex, age, total days of IV antibiotic, total days of oral antibiotic, total days of mechanical ventilation, duration of hospital and ICU stay, vasoactive and inotropic drug use, renal function, transfusion of blood products, and outcome. The Acute Physiology and Chronic Health Evaluation (APACHE) II disease classification system scores were calculated in all patients. Sequential Organ Failure Assessment (SOFA) score was also assessed on admission day.Infection severity was classified according to Sepsis-3 criteria: sepsis (life-threatening organ dysfunction caused by a deregulated host response to infection, suspected or overt infection, and acute increase of ≥2 points in SOFA scores in response to an infection, representing organ dysfunction) and septic shock (hypotension requiring vasopressors to maintain mean arterial pressure of > 65 mmHg and having a serum lactate level of > 2 mmol/L despite adequate volume resuscitation) [12]. Daily serum creatinine levels were assessed. Acute kidney injury (AKI) was classified according to the Kidney Disease Improving Global Outcomes criteria used in previous studies [13].

### Assessment and outcome

Daily oral and IV antimicrobial costs were calculated in all patients in local currency (Brazilian reals [BRL]). This was subsequently converted to United States dollars (US$), according to the official exchange values published by the Central Bank of Brazil, in the average of June 2018. The time of antimicrobial use, daily dose, indication, route of administration, and infection site were evaluated from admission in the ICU to discharge of the patient [14]. Antimicrobial costs between the OS group and non-OS group were calculated. The average costs of intensive care and direct daily ward care were calculated as follows: cost of consumables and drugs plus the cost of medical staff plus the cost of nursing and clinical support services, divided by the number of days in the respective units [15, 16]. The average daily ICU cost was US$ 393.09, and the average daily ward cost was US$ 86.31. The work sampling method was used in the evaluation of the nursing team activities (workload). The work was followed for 2 weeks for 3 shifts per day, taking into account only the time spent during the separation, dilution, and infusion of the antibiotics. The conversion to the financial value was performed by comparing the time spent by the hours-worked value [17].

After inclusion of the patient in the institutional sepsis protocol, culture-positive bacterial/fungal identification was performed using matrix-assisted laser desorption ionization-time of flight mass spectrometry, and the antimicrobial profile was determined by the VITEK 2 instrument. All preliminary results of the cultures of the biological materials were sent to the multiprofessional team. Crude mortality rate was calculated, a hospital overall survival curve was constructed, and ICU discharge was evaluated, comparing IV and OS therapy.

### Statistical analysis

Qualitative data were described as percentages, and quantitative data were described as arithmetic means or median values according to the distribution pattern. Standard deviation and 25 and 75% interquartile range (IQR) were the distribution variables for the mean and median, respectively. Risk factors associated with outcomes (death) were evaluated according to each variable, and distribution was determined using the Student’s t-test, Mann−Whitney, chi-square, or Fisher’s exact test. A difference of 5% (*p* < 0.05) indicated statistical significance. For the multivariate analysis, all variables with statistical significance in the univariate analysis (*p* < 0.05) were included in a binary logistic regression.

Survival curves (Kaplan−Meier) were constructed from the time of antibiotic initiation to the patient’s death or discharge. Overall mortality was included in the analysis, and 30-day mortality curves were constructed, and the Gehan−Breslow−Wilcoxon test was performed. All tests were performed using SPSS 23.

## Results

A total of 1313 patients were admitted to the two ICUs during the study period, and 349 patients fulfilled the sepsis and inclusion criteria.

Of the 349 patients included in the study, 142 (40.8%) fulfilled the criteria for sepsis without shock, and 207 (59.2%) presented with septic shock. Respiratory infection was the most frequently observed infection (*n* = 189, 54.3%), followed by abdominal infection (*n* = 45, 12.9%) and urinary infection (*n* = 38, 10.9%). The most prescribed antibiotics were cefepime (*n* = 122, 34.2%), ceftriaxone (*n* = 112, 32.2%), and aminoglycoside (*n* = 75, 21.6%) (Table 1). The most prevalent bacteria were *Staphylococcus aureus* (*n* = 20, 6%), *Escherichia coli* (*n* = 18, 5%), *Enterobacter* spp. (*n* = 16, 5%), *Klebsiella* spp. (*n* = 15, 4%), and *P. aeruginosa* (*n* = 13, 4%) (Table 2).

**Table 1:** Characteristics of patients in the oral switch stewardship program

Data	All (*n* = 349)		No oral switch (*n* = 238)		Oral switch (*n* = 111)		*P*-value	Odds ratio	Multivariable analysis
	N	%		%		%			
Male	208	59.7%	132	56%	77	69%	0.010	1.79 (1.11–2.89)	NS
Female	140	40.3%	106	45%	34	31%			
Heart failure class IV	41	12%	25	11%	16	15%	0.181		
Immunosuppression	27	8%	17	7%	10	9%	0.324		
Cirrhosis	7	2%	5	2.1%	2	1.8%	0.662		
Site of infection									
Respiratory	189	54.3%	122	51.3%	67	60.9%			
Urinary	38	10.9%	32	13.4%	6	5.5%			
Abdominal	45	12.9%	37	15.5%	8	7.3%			
Bloodstream	24	6.9%	22	9.2%	2	1.8%			
Skin and soft tissue	25	7.2%	11	4.6%	14	12.7%			
Central nervous system	4	1.1%	4	1.7%	0	0%			
Others	7	2.4%	0	0%	7	6.3%			
Undefined	15	4.3%	10	4.2%	5	4.5%			
Vasoactive drug									
Vasopressin	71	20.4%	62	26.1%	9	8.2%	< 0.001	0.25 (0.12–0.53)	NS
Noradrenalin	205	58.9%	137	57.6%	68	61.8%	0.264		
Dobutamine	153	44%	117	49.2%	36	32.7%	–		
Acute Kidney Injury	69	19.8%	54	22.6%	15	13.5%	0.032		NS
Antibiotic									
Aminoglycoside	75	21.6%	31	26.1%	22	11.8%	0.002	0.38 (0.19–0.72)	0.014
Polymyxin	12	3.4%	11	4.6%	1	0.9%	0.065		
Cefazolin	8	2.3%	1	0%	7	6.4%	0.002	16.1 (1.97–132.59)	0.004
Ceftriaxone	112	32.2%	82	34.5%	30	27.3%	0.113		
Cefepime	122	34.2%	82	34.5%	40	36.4%	0.409		
Carbapenem	39	11.2%	37	15.5%	2	1.8%	< 0.001	0.10 (0.02–0.42)	NS
Quinolone	77	22.1%	10	4.2%	67	60.9%	< 0.001	40.71 (19.76–83.89)	< 0.001
Vancomycin	95	27.3%	86	36.1%	9	8.2%	< 0.001	0.15 (0.07–0.32)	NS
SMX/TMP	24	6.9%	16	6.7%	8	7.3%	0.506		
Metronidazole/clindamycin	50	14.3%	37	15.5%	13	11.8%	0.226		
Macrolide	24	6.9%	15	6.0%	9	8.2%	0.332		
Penicillin	39		8	3.4%	31	28.2%	< 0.001	11.28 (4.97–25.56)	0.001
Mortality	44	12.6%	35	14.7%	9	8.2%	0.060		
Age	64 (53–73)		65 (55–74)		64 (51–73)		0.327		
APACHE II score	15.5 (12–19)		16.5 (13–19)		15 (14–17)		0.061		0.003
SOFA score	3 (2–5)		3 (2–5)		3 (2–4)		0.112		
IV antibiotic duration (days)	5 (4–7)		7 (5–10)		3 (2–4)		< 0.001		NS
Oral antibiotic duration (days)			0		4 (3–5)		–		
Mechanical ventilation (days)	3 (2–4)		3 (2–4)		3 (1.75–4)		0.008		NS
Total hospitalization (days)	13 (8–21)		13 (8–22)		13(8–20)		0.665		
Days in the ICU	6 (4–9)		6 (4–10)		5 (3–7)		0.029		NS

Penicillins – ampicillin/sulbactam; amoxicillin; amoxicillin/clavulanate

*SOFA* Sequential organ failure assessment, *IV* Intravenous, *ICU* Intensive care unit, *SMX/TMP* Sulfamethoxazole/trimethoprim, *APACHE* Acute physiologic assessment and chronic health evaluation

**Table 2:** Isolated bacteria by intervention group

	No oral switch (*n* = 238)		Oral switch (*n* = 111)		All (*n* = 349)		*P*-value
	N	%	N	%	N	%	
Negative	129	54%	76	69%	205	59%	0.024
Gram-negative	72	30%	17	15%			0.027
*Enterobacter* spp.	23	10%	4	4%	16	5%	0.139
Multisusceptible	7		4		11		
ESBL-producing	16		0		16		
*Escherichia coli*	14	6%	4	4%	18	5%	0.531
Multisusceptible	14		4		18		
*Klebsiella* spp.	12	5%	3	3%	15	4%	0.468
Multisusceptible	4		3		7		
Carbapenemase-producing	8		0		8		
*Pseudomonas aeruginosa*	11	5%	2	2%	13	4%	0.332
Multisusceptible	11		2		13		
*Serratia* spp.	6	3%	1	1%	7	2%	0.449
Multisusceptible	6		1		7		
*Acinetobacter baumannii*	0	0%	1	1%	1	0%	–
*Proteus* spp.	1	0%	1	1%	2	1%	0.433
Multisusceptible	1	0%	1	1%	2		
*Burkholderia cepacia*	2	1%	0	0%	2	1%	–
*Aeromonas hydrophila*	1	0%	1	1%	2	1%	0.433
*Citrobacter* spp.	1	0%	0	0%	1	0%	–
*Haemophilus* spp.	1	0%	0	0%	1	0%	0.433
Gram-positive	26	11%	15	14%			0.027
*Stenotrophomonas maltophilia*	1	0%	1	1%	2	1%	0.433
*Listeria monocytogenes*	1	0%	0	0%	1	0%	–
*CN Staphylococcus*	0	0%	1	1%	1	0%	0.433
*Staphylococcus aureus*	14	6%	6	5%	20	6%	0.372
MSSA	5	2%	3	3%	8	2%	
MRSA	9	4%	3	3%	12	3%	
*Streptococcus pneumoniae*	4	2%	4	4%	8	2%	0.097
*Enterococcus* spp.	4	2%	1	1%	4	1%	0.403
*Streptococcus* spp.	1	0%	1	1%	2	1%	0.433
Others							
*Pneumocystis jirovecii*	0	0%	1	1%	1	0%	–
*Candida albicans*	1	0%	0	0%	1	0%	–
Polymicrobial	2	1%	0	0%	2	1%	–
Total	238	100%	110	100%	348		

*ESBL* Extended-spectrum beta-lactamases, *MSSA* Methicillin-susceptible *Staphylococcus aureus*, *MRSA* Methicillin-resistant *Staphylococcus aureus*, *CN* Coagulase negative

In the OS group (*n* = 111), the mean age of patients was 60.9 years (median, 64 years; 25–75% IQR, 51–73), and 69% of patients were men. The mean length of ICU stay was 6.4 days (median, 5 days; 25–75% IQR, 3–7). The mean duration of hospitalization was 15.7 days (median, 13 days; 25–75% IQR, 8–20). The most prescribed antibiotics were quinolone (*n* = 67, 60.9%), cefepime (*n* = 40, 36.4%), and penicillin (*n* = 31, 28.2%) (Table 1). The most prevalent bacteria were *S. aureus* (*n* = 6, 5%) and *E. coli* (*n* = 4, 4%) (Table 2). The mean duration of oral antimicrobial treatment was 4.9 days (median, 4 days; 25–75% IQR, 3–5), and that of IV treatment was 3.4 days (median, 3 days; 25–75% IQR, 2–4).

In the non-OS group (*n* = 238), the mean age of patients was 62.98 years (median, 65 years; 25–75% IQR, 55–74). The most prescribed antibiotics were vancomycin (*n* = 86, 36.1%), ceftriaxone (*n* = 82, 34.5%), and cefepime (*n* = 82, 34.5%). The most prevalent bacteria were *Enterobacter* spp. (*n* = 23, 10%), *E. coli* (*n* = 14, 6%) and *S. aureus* (*n* = 14, 6%) (Table 2). The prevalence of multiresistant bacteria was insignificantly higher in the non-intervention group, with a predominance of extended-spectrum beta-lactamase-producing bacteria and MRSA. The frequency of negative cultures in the switch group was higher (*p* = 0.012) (Table 2).

**Fig. 1:**
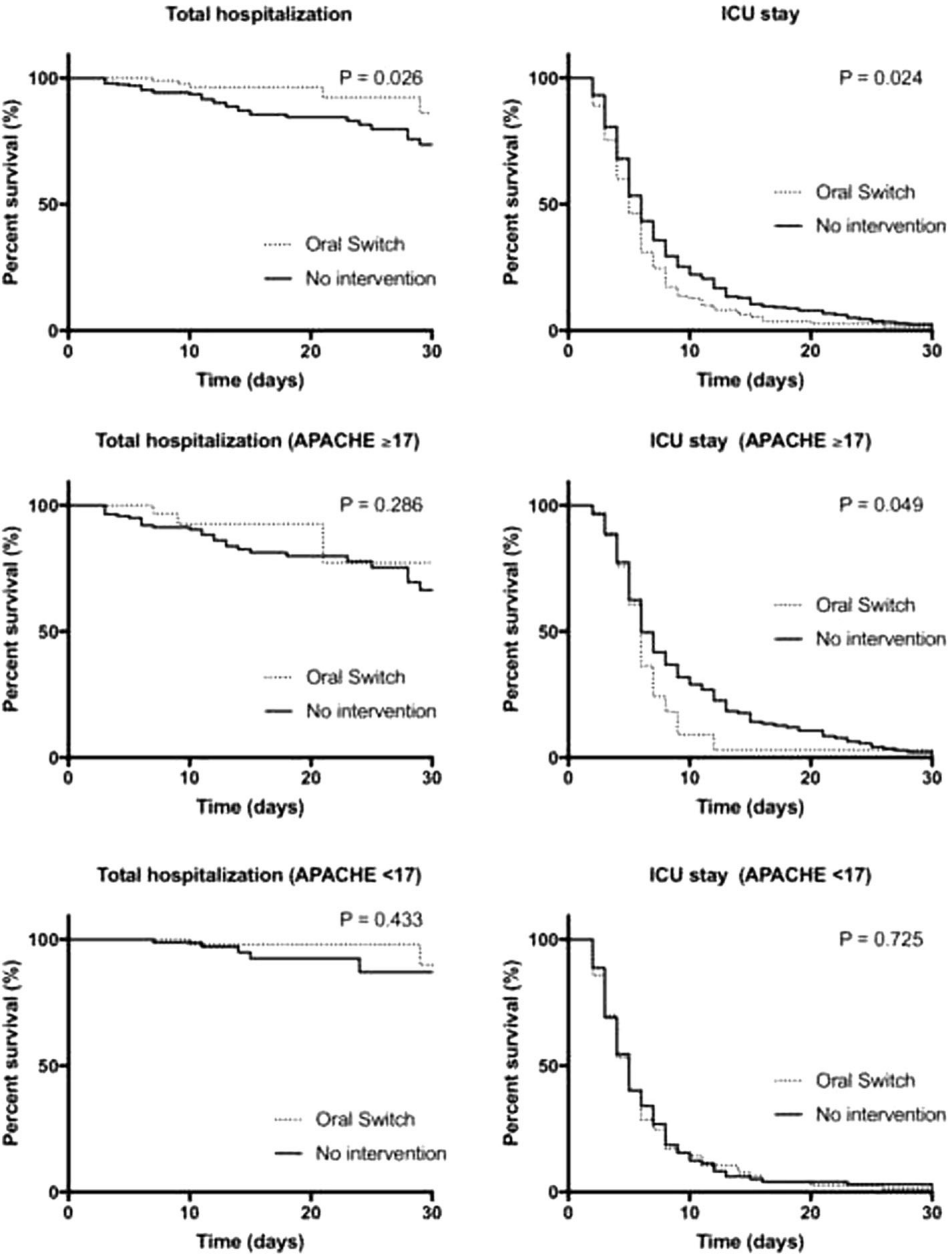
Survival and length of stay

The length of ICU stay was shorter in the OS group (*p* = 0.024), and the probability of survival in 30 days (*p* = 0.026) was determined using the Kaplan−Meier survival curve (Fig. 1). The severity of critical illness in the patients, ascertained by the APACHE II score (*p* = 0.061) and SOFA score (*p* = 0.112), was similar in the two groups in the univariate analysis. However, after removal of confounding variables, a significant difference was noted in both groups (*p* = 0.003). The confounding variables were excluded with the binary logistic regression (backward steps), which presented *p* < 0.05 in the univariate analysis, but not statistically significant in the multivariable analysis. However, there was no difference in global mortality between groups (*p* = 0.06). The APACHE II score was categorized using the ideal binning from SPSS 23 according to mortality. Patients in the OS group with an APACHE II score **≥** 17 presented a higher probability of survival (*p* < 0.05) (Fig. 1).

The direct costs of treatment, antibiotics, workload, and consumables were lower in the OS group (*p* < 0.001).

The average cost of antimicrobial treatment was lower in the OS group, with statistically significant differences in ICU costs, workload, and consumables (Table 3).

**Table 3:** Patient costs by intervention group

Costs	No oral switch		Oral switch		*P*-value
	Median	IQR (25–75)	Median	IQR (25–75)	
Ward	474.7	(172.6–1122.1)	517.9	(258.9–1122.1)	0.099
Total hospital	3010.6	(2090.3–5032.1)	2742.3	(2003.9–3847.2)	0.063
ICU	2358.5	(1572.3–3930.9)	1965.4	(1474.1–2849.9)	0.027
Antibiotics	22.7	(10.1–64.6)	10.2	(6.4–36.4)	< 0.001
Workload	8.6	(6.1–12.3)	3.6	(2.4–4.9)	< 0.001
Consumables	23.0	(2.7–52.3)	1.75	(0.8–4.9)	< 0.001

*ICU* Intensive care unit, *IQR* Interquartile range 25 to 75%

## Discussion

The present study aimed to evaluate a stewardship intervention based on the conversion of IV therapy to oral therapy as soon as clinical improvement was observed. The main data showed that there was no difference in mortality between the OS and non-OS groups, which makes this strategy safe for use in critically ill patients. In this population, the costs of antimicrobial treatment and daily intensive care value were lower in the OS group, although the total cost of hospitalization was not statistically different (*p* > 0.05).

Antibiotics account for a considerable cost in hospital bills, accounting for almost 20% of drug costs in our ICUs. The usage policy is a controversial subject, since it varies among hospitals, due to epidemiological differences, and among units from the same hospital, because it must consider the specificity of each location [18]. The total cost of hospitalization presented a reduction in cost of US$ 268.30 per patient but did not have statistical significance (*p* = 0.063). The reduction was not significant due to sample power in the OS group.

An antimicrobial stewardship program improves antimicrobial use in ICUs, resulting in shorter therapy durations, lower resistance rates, and lower costs [19]. Stewardship programs and a restrictive approach to antibiotic prescription can lead to several benefits, such as reduced cost, shortened length of stay, and reduced mortality rate. De-escalation of therapy and a switch from an IV to oral regimen, if it does not cause harm and demonstrates a safe strategy, can have an important outcome [20]. AKI was less frequent in patients who received OS. This could be attributed to several factors such as infection severity, aminoglycoside exposure, and cumulative dose of cephalosporin and vancomycin [21–23]. Oral drugs currently used in this study (quinolones and beta-lactams) are considerably less nephrotoxic than IV options (vancomycin and aminoglycosides). More than 20% of patients received aminoglycosides, and more than 30% received vancomycin in the non-OS group. There is a tendency toward higher costs in patients with AKI and undergoing dialysis, including longer hospitalization periods after sepsis [24]. Our study, although not showing a reduction in mortality rate, demonstrated a shortened time of IV therapy and shortened length of hospital stay, decreased antimicrobial use, and decreased incidence of AKI. Treatment of critically ill patients is usually initiated by IV administration. As demonstrated by Chin et al., administration of antimicrobial agents can be switched to the oral route when patients are able to tolerate oral intake, usually after 72 h of treatment. Patients who could not swallow tablets were administered an oral suspension via a nasogastric tube [25].

Rebuck et al. showed that levofloxacin is well absorbed after oral administration in critically ill patients in the ICU [5]. In our study, quinolones were one of the preferred classes of antimicrobial agents administered from IV to oral route in 60.9% of patients in this group. Several beta-lactams can be good alternatives in the conversion from IV to oral administration due to their high bioavailability [26]. In our study, this number corresponded to almost 30% of patients eligible for the oral route. IV-to-oral switch of antimicrobial therapy in the ICU is a controversial subject, as it depends on factors such as pharmacokinetics and pharmacodynamics of antimicrobial agents and clinical evolution of patients. As demonstrated by Rebuck et al. [5] and Carlier et al. [27], both quinolones and beta-lactams can be considered safe in IV-to-oral administration conversion in critically ill patients. In our study, 60.9% of the patients received quinolones, and 28.2% received penicillins in the OS group. These options were favorable considering the local microbiological profile of the units included in the study.

Even in critically ill patients with complicated abdominal infections, Solomkin et al. conducted the conversion of IV (imipenem) to oral therapy (quinolone). However, all benefits related to a shorter IV therapy treatment time, which would allow for earlier hospital discharge for some patients, and reduced drug and drug administration costs were unclear. Despite this fact, this study would support the efficacy of this approach, with low mortality rates (lower than 10%, as reported in our study) [28].

Antibiotic stewardship programs are safe and cost-effective, an approach important particularly in developing countries [29]. Our study demonstrated a difference in antimicrobial consumption at US$ 45.11 per day of treatment, with an estimated savings of US$ 13,947 between both groups (IV and oral). There was also a reduction of US$ 6,142 per patient of variable costs during ICU admission and US$ 1,800 in workload, with an estimated savings of US$ 514,831 as a result of the OS during the study period, even though the difference in length of ICU stay between groups was only 1 day. The difference in prescription between the two groups was influenced by the microbiological profile. The non-intervention group had an insignificantly higher prevalence of resistant bacteria, which limits the efficacy of oral antimicrobial therapy, despite the severity of critical illness between groups being similar. In contrast, it may be considered a benefit of the OS intervention to make the microbiological profile more favorable.

The present study showed some limitations, such as the type of the study (retrospective), inclusion of only 2 hospitals, use of a narrow range of antimicrobials, and specific microbiological profiles. A relevant factor was that the antimicrobial agent sulfamethoxazole-trimethoprim was not compatible with enteral catheter administration, which limited the sample size. This is an important issue considering that sulfamethoxazole-trimethoprim is the main alternative drug in our institution due to the susceptibility profile. In contrast, the data related to costs and doses of antimicrobials were individualized and not estimated by the defined daily dose, which would provide more reliable data on the consumption of antimicrobials. The sample of patients subjected to OS has in itself different characteristics, generally of lower clinical severity and lesser bacteriological complexity. Some pharmacokinetic aspects can be different in oral administration, as they can be different in patients with sepsis. OS is part of the antimicrobial stewardship program; thus, it is impossible to attribute all influences of these interventions on the final results.

## Conclusion

Stewardship programs based on OS therapy in ICUs can be a safe intervention, thereby decreasing the cost and shortening the length of stay when well-defined criteria are employed and a clinical decision is reached. In developing countries, where limited resources and intensive care beds have a major impact on resource management, such a strategy can have a positive contribution. Randomized trials with a larger number of patients are required to clarify the large-scale impact of this strategy.

## Additional file

Additional file 1 : STROBE Statement—checklist of items that should be included in reports of observational studies. (DOC 78 kb)

## Data Availability

Data are available under request. Please contact Dr. Felipe Tuon: felipe.tuon@pucpr.br

## Abbreviations

APACHE: Acute Physiology and Chronic Health Evaluation
ICU: Intensive care unit
IQR: Interquartile ranges
MRSA: Methicillin-resistant *Staphylococcus aureus*
SOFA: Sequential Organ Failure Assessment
