# Supplementary material for: Intravenous-to-oral antibiotic switch therapy: a cross-sectional study in critical care units
Source: BMC Infect Dis. 2019 Jul 22;19:650. doi: 10.1186/s12879-019-4280-0 (PMC6647098; doi:10.1186/s12879-019-4280-0)
Supplement: Supplementary file 1 — : STROBE Statement—checklist of items that should be included in reports of observational studies. (DOC 78 kb) [file 12879_2019_4280_MOESM1_ESM.doc]

STROBE Statement—checklist of items that should be included in reports of observational studies

|  | Item No | Recommendation |
| --- | --- | --- |
| **Title and abstract** | 1 | (*a*) Page 1 |
|  |  | (*b*) Page 2 |
| Introduction | | |
| Background/rationale | 2 | Page 3 |
| Objectives | 3 | Page 4 |
| Methods | | |
| Study design | 4 | Page 5 |
| Setting | 5 | Page 5 |
| Participants | 6 | (*a*) Page 6 |
|  |  | ( |
| Variables | 7 | Page 7 |
| Data sources/ measurement | 8* | Page 7 and 8 |
| Bias | 9 | Page 8 |
| Study size | 10 | N/A |
| Quantitative variables | 11 | Page 8 and 9 |
| Statistical methods | 12 | Page 9 |
|  |  | Page |
|  |  | Page |
|  |  | Page |
|  |  | Page |

Continued on next page

| Results | | |
| --- | --- | --- |
| Participants | 13* | (a) Page 10 |
|  |  | (b) Page 10 |
|  |  | (c) N/A |
| Descriptive data | 14* | (a) Page 10 |
|  |  | (b) Page 10 |
|  |  | (c) N/A |
| Outcome data | 15* | *Cohort study*— N/A |
|  |  | *Case-control study—*N/A |
|  |  | *Cross-sectional study—* Page 10, 11, 12, 13 |
| Main results | 16 | (*a*) Page 12, 13 |
|  |  |  |
|  |  |  |
| Other analyses | 17 | N/A |
| Discussion | | |
| Key results | 18 | Page 14 |
| Limitations | 19 | Page 15 |
| Interpretation | 20 | Page 14, 15 |
| Generalisability | 21 | Page 14, 15 |
| Other information | | |
| Funding | 22 | N/A |

*Give information separately for cases and controls in case-control studies and, if applicable, for exposed and unexposed groups in cohort and cross-sectional studies.

**Note:** An Explanation and Elaboration article discusses each checklist item and gives methodological background and published examples of transparent reporting. The STROBE checklist is best used in conjunction with this article (freely available on the Web sites of PLoS Medicine at http://www.plosmedicine.org/, Annals of Internal Medicine at http://www.annals.org/, and Epidemiology at http://www.epidem.com/). Information on the STROBE Initiative is available at www.strobe-statement.org.
